# Implementing electronic data capture at a well-established health and demographic surveillance site in rural northern Malawi

**DOI:** 10.1080/16549716.2017.1367162

**Published:** 2017-09-18

**Authors:** Estelle McLean, Albert Dube, Jacky Saul, Keith Branson, Mabvuto Luhanga, Oddie Mwiba, Fredrick Kalobekamo, Steffen Geis, Amelia C Crampin

**Affiliations:** ^a^ Malawi Epidemiology and Intervention Research Unit, Karonga, Malawi; ^b^ Faculty of Epidemiology and Population Health, London School of Hygiene and Tropical Medicine, London, UK

**Keywords:** Electronic data capture, Health and Demographic Surveillance Site, Malawi, data quality

## Abstract

This article aims to assess multiple issues of resources, staffing, local opinion, data quality, cost, and security while transitioning to electronic data collection (EDC) at a long-running community research site in northern Malawi. Levels of missing and error fields, delay from data collection to availability, and average number of interviews per day were compared between EDC and paper in a complex, repeated annual household survey. Three focus groups with field and data staff with experience using both methods, and in-depth interviews with participants were carried out. Cost for each method were estimated and compared. Missing data was more common on paper questionnaires than on EDC, and a similar number were carried out per day. Fieldworkers generally preferred EDC, but data staff feared for their employment. Most respondents had no strong preference for a method. The cost of the paper system was estimated to be higher than using EDC. The existing infrastructure and technical expertise could be adapted to using EDC, but changes have an impact on data processing jobs as fewer, and better qualified staff are required. EDC is cost-effective, and, for a long-running site, may offer further savings, as devices can be used in multiple studies and perform several other functions. EDC is accepted by fieldworkers and respondents, has good levels of quality and timeliness, and security can be maintained. EDC is well-suited for use in a well-established research site using and developing existing infrastructure and expertise.

## Background

Electronic data collection (EDC) has become common in low-resource settings, and has been useful in many types of health research, including large one-off household surveys [1], on-going clinic-based studies [2], and in long-term community field sites [3]. Introducing EDC to a rural research site with existing long-running protocols requires several issues to be considered:

1. *Availability of appropriate hardware and software*: Devices have improved in quality in recent years, but in low-resource settings sourcing and maintaining equipment can be difficult, due to lack of local services [4]. Inconsistent electricity also causes problems with keeping devices charged in rural locations [2,5]. Several data collection software programmes now exist.
2. *Technical expertise required*: In rural low-resource settings, finding local staff with the higher level of technical skills required maybe challenging.
3. *Cost*: While economic analyses have found that, assuming a study beyond a certain size, the costs tend to be lower with EDC [6,7], a long-running site with existing infrastructure may incur different costs and savings.
4. *Acceptance of staff*: It has been found that lack of previous experience with computers has not been a hindrance to fieldworkers learning to use the devices [2,8], and users tend to prefer EDC over paper [8–13]. However, fieldworkers are not the only people affected by a change to established procedures.
5. *Acceptance of the respondents*: Long-running sites rely on maintaining good relationships with the local community. Where studied, respondents tended to have accepted EDC methods [8,] but in some place concerns were raised over ‘outsider’ technology [4,14].
6. *Data quality*: Systems of double-entry, checks, and verification ensure a high level of data quality in a paper-based system, and EDC must meet the same standards: most evaluations found that EDC was less prone to errors than paper [3,6,10,11,15–18].
7. *Time required*: Changes in the time needed for certain activities will have knock-on effects on other procedures, so it is important to assess these differences, and whether procedures can be adapted. EDC has generally been found to be more time-efficient compared to paper-based methods [3,7,19].
8. *Data security*: Security is important for any research study, especially if collecting potentially sensitive health information. EDC has different security issues from paper: losses of devices and data due to theft have been reported [2,8]; however software can be used to make EDC secure on the device [2].

While many studies have evaluated EDC in low-resource settings for health research, most tend to focus on the quality of the data, and few have described the introduction of such techniques on operational considerations in a long-running multi-study research site.

## Objective

This mixed methods study assessed the above eight issues while transitioning to EDC methods at the Karonga Health and Demographic Surveillance Site (HDSS) in northern Malawi.

## Methods

### Study setting

The Karonga HDSS was established in 2002 and captures births, deaths, and in- and out-migrations in a population of about 39,000 people living in a 150 km^2^ rural area [20]. All deaths are assessed using a verbal autopsy, and information on each individual’s socio-economic status is collected annually. The HDSS is used as a platform for health-related research, and five to 10 studies are conducted concurrently at any one time.

Data are collected from respondents at their homes or at clinics by fieldworkers. Since the initiation of the surveillance, paper forms have been used, which are double-entered into Microsoft Access databases by data processors. Verification of double-entered data is performed daily, and data are accessible to scientists and data managers 1 day after they are verified.

EDC was first introduced at the site in 2013: two new cross-sectional surveys were conducted using Open Data Kit (ODK) software (opendatakit.org) on android tablets and smartphones. After this demonstration of the utility of the technology in the area, and with additional grant support from the Wellcome Trust, it was decided to switch some long-running studies to EDC. In 2014, the verbal autopsy and, in 2016, the annual socio-economic survey were switched to EDC, both using ODK on android tablets. As other studies are still using paper, both paper-based and EDC systems are running concurrently.

### Quantitative methods

A validation exercise comparing the quality of data collected on paper and EDC was carried out using the socio-economic survey. All field-team members were already familiar with the questionnaire and were trained to use tablets. The team was split randomly: half started using EDC immediately, while the rest of the team continued to use paper forms, which were double-entered as per the established protocol. After 4 weeks of implementation of EDC, interviews carried out over a 3-week period in July 2016, by interviewers who had already done at least 20 interviews on either EDC or paper, were assessed in the following ways:

- Missing data: missing data was defined as not asked (blank; discounting ‘not applicable’ blank questions), or blank and entered as unknown combined (as most fields are required on EDC so cannot be blank). The proportion of missing data was compared using risk ratios, overall, and by the complexity of the skip pattern: filled depending on the answers to 0, 1, 2, or 3 or more previous questions.
- Internal validity: an error was defined as a field with an impossible or inconsistent value; the proportion of data errors were compared using risk ratios.
- Time from interview to data available on the database: The mean time between date of interview and entry/upload or edit date (which ever was later) was compared between the two groups.
- Average number of interviews per interviewer per day were compared.

### Qualitative methods

Three focus groups with staff members were carried out in November 2016. Fifteen fieldworkers and 14 data processors were included, nine (31%) were female, the median age was 31 years (range = 20–48 years), the median years of employment with the organisation was 5 years (range = 1–22 years), all had at least a secondary school leaving certificate (MSCE), with eight (30%) with additional certificates (MSCE+) and seven (26%) a further diploma. The focus groups were carried out in English, led by an experienced qualitative researcher using a topic guide including opinions and experiences of EDC and paper data collection and effects on individual jobs and the organisation. In February 2017, in-depth interviews were carried out with 10 purposively sampled community participants who had previously been interviewed with both paper and EDC methods. Four were female, and the median age was 32 (range = 19–67 years). Each interview lasted about 15 minutes, and was carried out in the local language (Tumbuka) by two experienced researchers in the participant’s home. All focus groups and interviews were audio-recorded, and notes made, which were used for analysis. Two researchers coded the data manually into broad and minor themes.

Additionally, senior staff involved in programming, IT, programme management, and data collection supervision were invited to share their experiences and opinions on the software, hardware, and logistics. Their responses were collated and summarised.

### Costing estimation

For a simple cost-comparison, the procedures used for paper and EDC for one round (12 months) of the socio-economic survey were compared. The costs for the stages that were different were estimated, and the overall cost differences compared.

### Ethics approval

The socio-economic study, nested within the demographic surveillance study, was approved by the Malawian National Health Sciences Review Committee (#419) and the London School of Hygiene and Tropical Medicine ethics board. Participation in the DSS and associated studies requires written informed consent.

## Results

### Quantitative

A total of 1161 interviews were carried out during the 3-week period by eight interviewers using paper and eight using EDC. In total, 177 interviews were excluded from the analysis when the interviewer had switched to the other method for all or part of a day, leaving a total 984 interviews; 426 on paper, and 558 on EDC.

#### Mean proportion of missing data

Overall, 492 (2.2%) of 21,976 fields on paper data forms were missing (blank), compared to 153 (0.7%) of 21,937 EDC fields, giving a risk ratio (RR) of 3.2 (95% CI = 2.7–3.8); including data entered as unknown reduced the RR to 2.2 (1.9–2.5) (Table 1). On paper forms, the level of missing data increased with the complexity of the skip pattern, from 0.1% of fields with no skip pattern to 3.9% of fields where the skip pattern depended on the answers to three or more previous questions. On EDC, this pattern was different: the level of missing data in fields dependent on just one previous question was higher than on more complex questions. However, at 1.1% (91 of 8064 fields), it was still lower than for the same variables on paper: 204 of 8148 fields, 2.5%, RR = 2.2 (1.7–2.8); including data entered as unknown reduced the RR to 1.4 (1.2–1.7) (Table 1).

**Table 1.** Missing data comparison between paper and EDC, overall, and by complexity of skip pattern.

Variable type		Overall	No skip	Skip dependent on 1 previous question	Skip dependent on 2 previous questions	Skip dependent on 3 or more previous questions
Total fields	Paper	21,976	5094	8148	6803	1931
	EDC	21,937	5022	8064	7001	1850
Missing fields	Paper	492 (2.2%)	4 (0.1%)	204 (2.5%)	209 (3.1%)	75 (3.9%)
	EDC	153 (0.7%)	0 (0.0%)	91 (1.1%)	62 (0.9%)	0
	RR	3.2 (2.7–3.8)	—	2.2 (1.7–2.8)	3.5 (2.6–4.6)	—
Missing and unknown fields	Paper	611 (2.8%)	4 (0.1%)	271 (3.3%)	246 (3.6%)	89 (4.6%)
	EDC	282 (1.3%)	0 (0.0%)	191 (2.4%)	87 (1.2%)	2 (0.1%)
	RR	2.2 (1.9–2.5)	—	1.4 (1.2–1.7)	2.9 (2.3–3.7)	42.6 (10.5–172.9)

#### Mean proportion of data errors

Very few internal inconsistencies were found; nine (0.2%) of 3622 fields on paper and 19 (0.5%) of 3590 on EDC, RR = 0.5 (0.2–1.1).

#### Time from interview to data available on the database

There was a mean of 3.4 days (3.0–3.7) between data collection and availability for paper data, this was lower for EDC at 2.1 days (2.0–2.3).

#### Average number of interviews per day

The mean number of interviews per day was similar for the two groups, at 10.7 (8.7–12.6) on paper and 11.8 (8.1–15.5) with EDC.

### Qualitative

#### Staff focus group discussions

The advantages and disadvantages of using EDC according to fieldworkers and data processors are shown in Table 2.

**Table 2.** Summary of user observed advantages and disadvantages of EDC.

Positive	Negative	Neutral/mixed
*Data collection*		
Easier to carry Skip patterns, entry constraints, and required fields Easier to change response Enthusiasm of respondents	Hard to switch between interviews Interview takes longer Tablets freeze occasionally Respondents might think they are being recorded Can forget to fill other forms without paper to remind you	Both EDC and paper are easy to use and required training Electronic device is smaller and easier to protect, but damage more severe if rain gets in
*Data management*		
Printing time reduced Data more secure Data available for analysis sooner	Easier to scan through paper form to check	Mixed views on data quality
*Other*		
Improved fieldworkers job Institution seen as more successful	Added task of charging and distributing electronic devices each day Electronic devices prone to theft and damage Data office jobs at risk	Electronic devices expensive but save on paper and printing costs

##### Data collection

Fieldworkers appreciated not having to carry and organise lots of papers. The interactive capabilities of EDC, such as automatic skip patterns and required fields, were felt to be improvements. Many fieldworkers felt that EDC interviews took longer, and occasionally devices froze during an interview. However, fieldworkers found it easier to engage with respondents, who they felt were enthusiastic about the new technology:

‘people ... have confidence in us, if these people bring these expensive items into the field it means that they are serious’ (M31, Field, MSCE+).

The absence of physical paper forms forced some changes in working practices that staff were used to, such as being able to switch between interviews quickly and having an easy reminder of other tasks to do after the interview. The knowledge that the data would not go through as many checks before being uploaded, and being trusted to use an expensive device, gave fieldworkers a greater sense of pride and responsibility.

… before [using paper] I knew that if I make any mistake the data officer will come back to me … with the tablet you feel more responsible, as if I make any mistake here it goes straight into the server (M22, Field, Diploma).

Staff found both methods easy to use, and felt that, with training, anyone could do either.

##### Data management

Data was felt to be more secure on the password-protected devices. However, losing or breaking a tablet was thought to have greater repercussions than losing a paper form, as other data could be lost, plus the cost of repairing or replacing the device.

Fieldworkers mostly believed that data quality was increased by using EDC, whereas data processors generally believed the opposite.

I think [we] will have a lot of false data compared to before … we find a lot of errors on the paper … we go back to the fieldworkers to verify … with the electronic data there will be no such back and forth … you just upload ... it won’t be quality data (F38, Data, MSCE+).

While the elimination of printing questionnaires and entering data was felt to be positive, as it decreased costs and made data available for analysis more quickly, there was also fears of job losses among data processors. Data processors involved in EDC had seen their responsibilities change, but there were no concerns over the content of the job, only that the job existed:

‘there was also that fear that we are going to lose our jobs … and a lot of rumours … that the data office will be reduced’ (M32, Data, Diploma).

##### Preference

Despite fairly balanced numbers of advantages and disadvantages, most staff members, including all fieldworkers, stated a preference for EDC over paper. The main reason for this was the feeling of ‘moving with the times’, which was felt to be good for the individual staff members, the standing of the organisation, and the country. Some data officers preferred to use only paper-based methods, mainly due to concerns over job losses and potential reductions in data quality.

#### View of respondents

Community respondents were largely indifferent to the method of data collection, and trusted the institution to choose the best method. Some felt that interviews were quicker when using the tablet, and some people imagined the device to be more robust, making data less likely to be lost or more resistant to rain than paper:

‘Using paper interviewers take a long time while when they use tablets … it is just like a computer’ (F19); ‘A tablet was made to simplify things. You can just connect this to other machines and same time you have [the] information’ (M33).

#### Senior staff reflections

##### Software

We decided not to use OpenHDS, a system specifically for HDSSs, as we wanted to use only one system for all studies across the site. We chose ODK as it is free, does not require an internet connection, and it is relatively easy to design forms. ODK allows for programming of the questionnaire in multiple languages and to toggle between them, which is useful, as several languages are in common usage in Malawi. To avoid interview fatigue, certain answers from the previous year were pre-printed on the paper form of the socio-economic survey for the interviewer to skip, or check and edit. It is possible to mimic this with ODK; however, it requires some time to download the data onto the tablets and causes a small delay when opening new forms. It may also contribute to the tablet ‘freezing’ occasionally experienced. ODK does not currently allow for editing a form which has already been uploaded onto the server. We felt that large amounts of text would take too long to key into the devices; in the few instances where interviewers need to make a longer note, ODK allows for alerts to prompt interviewers to fill a paper sheet, then to photograph it at the appropriate point in the questionnaire.

Although ODK is simple to use, some level of skill is required to create the forms. An external organisation was contracted to create forms for the first survey, but in-house development by staff with knowledge of field operations was preferable, particularly as turnaround on alterations and corrections of errors was much faster. Programming to ‘load’ the data from the server to the database was also developed in-house.

The lockdown software SureLock (https://www.42gears.com/products/surelock/) is used, which ensures that devices are not used for any other purpose.

##### Hardware

We used Toshiba AT10 and AT300 10” tablets and Samsung S3 GT i9300 4.8” smartphones which were purchased in the UK as prices were higher in Malawi. Despite using cases, 11 of 92 tablets broke over 4 years and had to be replaced (as screen replacement, the most common breakage, exceeded device cost). Theft was uncommon: only one functioning tablet and one smartphone went missing over the 4-year period. The existing secured wireless network was already sufficient for uploading data from the tablets to a locally installed ODK Aggregate server.

##### Logistics

Battery life has been adequate for using all-day in the field, although some devices have had problems as they aged. A dedicated, secured area was created for devices to be charged overnight each day: this was feasible as the campus is on the national grid with generator back-up, and fieldworkers start and end their working day at this base.

Use of EDC has required a change of study design culture. Scientists designed paper questionnaires independently (whilst following standardised layouts and conventions) and shared them with programmers at a relatively late stage; with EDC, engagement with programmers is much earlier.

Checks and processes are carried out manually on paper forms by data processors before data entry, including assigning identifying numbers to people and houses, and checking whether newly-reported data should be used to correct previously recorded information on sex and/or birth-date. Having first contact with data in electronic format has meant that these manual processes can now only be carried out by data processors with additional computer training.

#### Costs

The procedures for each method are listed in Table 3; the main differences were in printing the questionnaires and double-entering the data. In the most recent 12-month round of the survey, 41,050 two-page forms were filled: printing took two people 2 days per group (21 groups in total) and was replaced by a much quicker process of loading data to each of the tablets, taking one person 1 hour per group; data entry took an average of 4 minutes per form, and verification of the double-entered data happened daily, which could take 3 hours, this was replaced by one process to load the data from the server to the database, which is carried out by one full-time experienced data processor. In total, the estimated costs for the stages that are unique to the paper-based process are £18,895 per annum, which is 65% higher than the unique costs for the EDC system, £11,427 (Table 3).

**Table 3.** Cost estimates for paper or EDC methodology for the 12-month socio-economic study; similar or identical procedures are greyed out and costs not estimated.

Paper-based data collection			Electronic data collection		
Stage	Activity/item	Cost	Stage	Activity/item	Cost
Design questionnaire			Design questionnaire		
			Develop & test EDC form	Senior data manager time	£1,400
Develop data tables			Develop data tables		
Develop entry screens	Senior data manager time	£1,400	Develop load programme	Senior data manager time	£700
Pilot questionnaire			Pilot questionnaire		
Create data collection & entry protocols			Create data collection & entry protocols		
Train field & data staff			Train field & data staff		
Print questionnaires	Data officer time (2 people 2 days per group = 42 days)	£835	Load data to devices	Devices (16 devices @ £150)	£2,400
	Paper cost (166 reams @ £3.50)	£581		Data officer time (1 person 1 hour per group = 3 days)	£60
	Printer cost (12 cartridges @£198)	£2,376			
Collect data			Collect data		
Check & submit data to data office			Check & upload data to server		
Enter data (double)	Data officer time (4 minutes per form × 2)	£9,691	Load data to database	Data officer time (1 person full-time)	£6,868
Verify double data entry	Data officer time (1 person 3 hours per day)	£1,590			
Store paper forms	Librarian time (1 minute per form)	£2,423			
Check & clean data			Check & clean data		
Analyse data			Analyse data		
	Total:	£18,895		Total:	£11,427

## Discussions

In this mixed methods evaluation of EDC at a well-established research site in rural Malawi, we found EDC to be useful according to the following criteria:

### Availability of appropriate hardware and software

The devices we used have largely met our expectations and requirements, apart from some isolated reports of tablets freezing, which has been found in other settings [8,12]. Existing site infrastructure meant that some challenges were easier to overcome, for example keeping devices charged, which has been shown to be a difficulty elsewhere [2]. As with other groups in Malawi, we found ODK to be useful, as it functions without internet connectivity [8]. The ODK software provides most of the functions that we required, but certain things, like being able to edit a form once it has been finalised, are currently not possible. Other research sites, including HDSSs, have developed their own software for such purposes [1,21,22], which was beyond our current capacity.

### Technical expertise required

We found it more efficient to develop ODK forms in-house, and existing staff members were able to develop skills in that area. Developing programs to load data from the ODK server to databases requires specific skills, already available to us. Although our site is based in a rural location in a resource-limited country, international staff employed through the London School of Hygiene & Tropical Medicine in the UK provided most of the technical expertise. To implement a more complex system would have required additional expertise not yet available.

### Cost

We estimate that, for our large household study, paper-based methods were more expensive than using EDC. With a study size above a certain threshold (as start-up costs can be high), costs tend to be lower with EDC [6,7,10,13]. We were able to reduce costs in some areas, for example by buying devices in the UK, as they were expensive to buy locally. Devices can be re-used for different studies and, as they are able to perform multiple functions, additional devices may not be needed to be purchased in future, such as GPS recorders, voice recorders, and digital cameras. Additionally, costs may be saved on storing paper forms. Switching to EDC at our site was made possible by a specific grant.

### Acceptance of staff

In general, EDC had a positive effect on fieldworkers’ job satisfaction. Other commentators also reported that users tended to prefer EDC over paper [8–13] and that interviewers without prior computing experience readily adapted to tablet use [2,8,23]. The experience has been less positive for data processing staff who feared for employment security, especially those without the higher level of skills and experience required to work on EDC.

### Acceptance of the respondents

While fieldworkers reported that using EDC increased engagement of respondents; the respondents themselves were actually non-committal on the method of data capture. In other evaluations, respondents tended to have accepted the new methods [8,23], but some concerns were raised over ‘outsider’ technology [4,14], and one study found a higher refusal rate with EDC [15]; the respondents we spoke to had no reservations regarding the technology. This acceptance in our setting may be due to the long-running nature of the project and good relationships with the community.

### Data quality

Interviews carried out using EDC had a lower proportion of missing data, and a similar level of internal validity, compared to those collected on paper. Our quantitative results on data quality are consistent with findings from other evaluations [3,6,7,10,11,15–19]. On paper forms, the level of missing data increased with the complexity of skip patterns, while almost the opposite pattern was seen in EDC, showing the usefulness of programmed automatic skip patterns in guiding interviewers to the correct questions. On EDC, the questions dependent on just one previous question had the highest level of missing data; on our questionnaire these questions were mostly starting questions for sections asked to people dependent on their age, so missing data could lead to a whole section being missed. The level of missing data in these questions was still lower in EDC than paper, potentially due to ‘prompts’ we pre-emptively programmed, which remind fieldworkers that certain sections will not be displayed due to data they had entered. Our observations on the quality of EDC data contrasts with the opinion of some data processors, who felt that data quality would be compromised by foregoing human checks. This discrepancy may be due to the lack of familiarity of the data processors with EDC functions and the programmed checks.

### Time required

The daily average number of interviews was similar for paper and EDC. We did not measure the length of each interview, but fieldworkers generally thought that EDC interviews took longer, while respondents felt the opposite. Misperception about interview length has been observed elsewhere [15], and may be due to the delays on loading and saving forms being more apparent to fieldworkers than delays incurred while using paper (for example, manually navigating the skip patterns). Using EDC reduced the time needed before and after data collection, as printing and data entry were largely eliminated; the latter reflected in the reduction in the average number of days from data collection to availability to scientists. Other evaluations have found that the overall time required was always shorter with EDC [3,6,7,13,19], despite additional time needed at the beginning for making tablet forms [5,15], which is consistent with our findings.

### Data security

Fieldworkers were not concerned over being targets for thieves while carrying devices, and we experienced very little theft; crime is generally low in this area. Both fieldworkers and data processors felt that data would be more secure on the tablet, due to the password protection. As we did not need to rely on mobile phone networks, and our existing local area network was already secure, we were satisfied that using EDC would not pose a risk to data security.

A strength of this study has been being able to document the views of research staff and respondents with long-term experience of both paper and EDC methods. However, the process of switching to EDC is not complete for all studies, and other issues may arise. A limitation of our quantitative analysis was only being able to assess a few variables for potential errors or internal consistency, rather than being able to compare data collected on each method to a ‘gold standard’, as in some evaluations which used simulation techniques such as using made-up data or educated respondents with repeat interviews using the different methods [3,19]. However, it is equally important to test these methods in real-world settings. We were not able to do a full-economic analysis and were not able to assess the environmental impact: it is not clear whether reducing consumption of paper and printer ink is offset by device production, delivery, and disposal. Our findings are specific to the setting, although many of the results should be generalizable to other settings.

## Conclusion

Considering multiple issues of resources, staffing, local opinion, data quality, cost, and security, EDC is well-suited for use in a well-established research site, using and developing existing infrastructure and expertise. Adapting EDC to established data processing procedures may, however, require more complex solutions and any changes, particularly if they have an impact on job descriptions and employment security should be managed appropriately.

## Paper context

Electronic data capture (EDC) has been found to give high quality data in low-resource settings; however, few have described its impact beyond data quality or in long-running multi-study research sites. We show that EDC can be successfully implemented in well-established sites such as ours in Northern Malawi. Sites considering switching to EDC should be aware that more complex solutions may be required to fit with existing protocols and negative effects on staff should be anticipated.
